# Spontaneous Structural Changes in Actin Regulate G-F Transformation

**DOI:** 10.1371/journal.pone.0045864

**Published:** 2012-11-05

**Authors:** Masatoshi Morimatsu, Yuichi Togashi, So Nishikawa, Mitsuhiro Sugawa, Atsuko H. Iwane, Toshio Yanagida

**Affiliations:** 1 Nanobiology Laboratories, Graduate School of Frontier Biosciences, Osaka University, Suita, Osaka, Japan; 2 Department of Computational Science, Graduate School of System Informatics, Kobe University, Kobe, Hyogo, Japan; 3 Quantitative Biology Center (QBiC), RIKEN, Suita, Osaka, Japan; Karolinska Institutet, Sweden

## Abstract

Transformations between G- (monomeric) and F-actin (polymeric) are important in cellular behaviors such as migration, cytokinesis, and morphing. In order to understand these transitions, we combined single-molecule Förster resonance energy transfer with total internal reflection fluorescence microscopy to examine conformational changes of individual actin protomers. We found that the protomers can take different conformational states and that the transition interval is in the range of hundreds of seconds. The distribution of these states was dependent on the environment, suggesting that actin undergoes spontaneous structural changes that accommodate itself to polymerization.

## Introduction

Actin, one of the most abundant proteins in eukaryotes, plays a key role in many cellular processes such as migration and morphing by switching between its monomeric (G-actin) and polymeric (F-actin) forms [1]. The architectures of the actin network affect the elasticity of the cell [2]. While the crystallographic structure of G-actin has been solved in various conditions [3], [4], [5], [6], the equivalents for F-actin are not known. Structural models of F-actin were constructed on the basis of the G-actin structures [7], [8]. Recent cryo-electron microscopy (cryo-EM) studies have suggested structural models for F-actin; one of them showed a single well-defined structure [9], [10], and another suggested structural polymorphisms [11].

Actin polymerization proceeds when the concentration of G-actin exceeds a critical concentration, which is defined as the concentration of monomers that achieves a dynamic equilibrium for actin filament polymerization and depolymerization. The critical concentration is high in the absence of salts, but very low at physiological concentrations. F-actin undergoes actin treadmilling, a phenomenon where the filament length remains approximately constant despite actin monomers attaching to the plus end (barbed end) of the filament and dissociating from the minus end (pointed end). Although G-F transformation can be regulated by a variety of actin-binding proteins (ABPs) at physiological conditions [12], recent data detailing the structures suggest that G-F transformation involves large conformational changes in actin protomers independent of ABP [13]. Furthermore, the existence of an intermediate state of G-actin preceding the transformation in solution, called F-monomer [14], G*-actin [15], and KCl-monomer [16], have been suggested.

F-actin in solution has high flexibility [17] and the structure is affected by ABPs (e.g., twisting upon binding of cofilin [18]). However, the corresponding conformational dynamics of actin are only suggested on the basis of static structural models; the actual dynamics in solution remain poorly understood. Therefore, to better understand the fundamental properties of these dynamics, we here report the direct observation of single G- and F-actin protomers using single-molecule Förster resonance energy transfer (smFRET) in combination with total internal reflection fluorescence (TIRF) microscopy.

## Results

### Conformational states of G-actin

**Figure 1:**
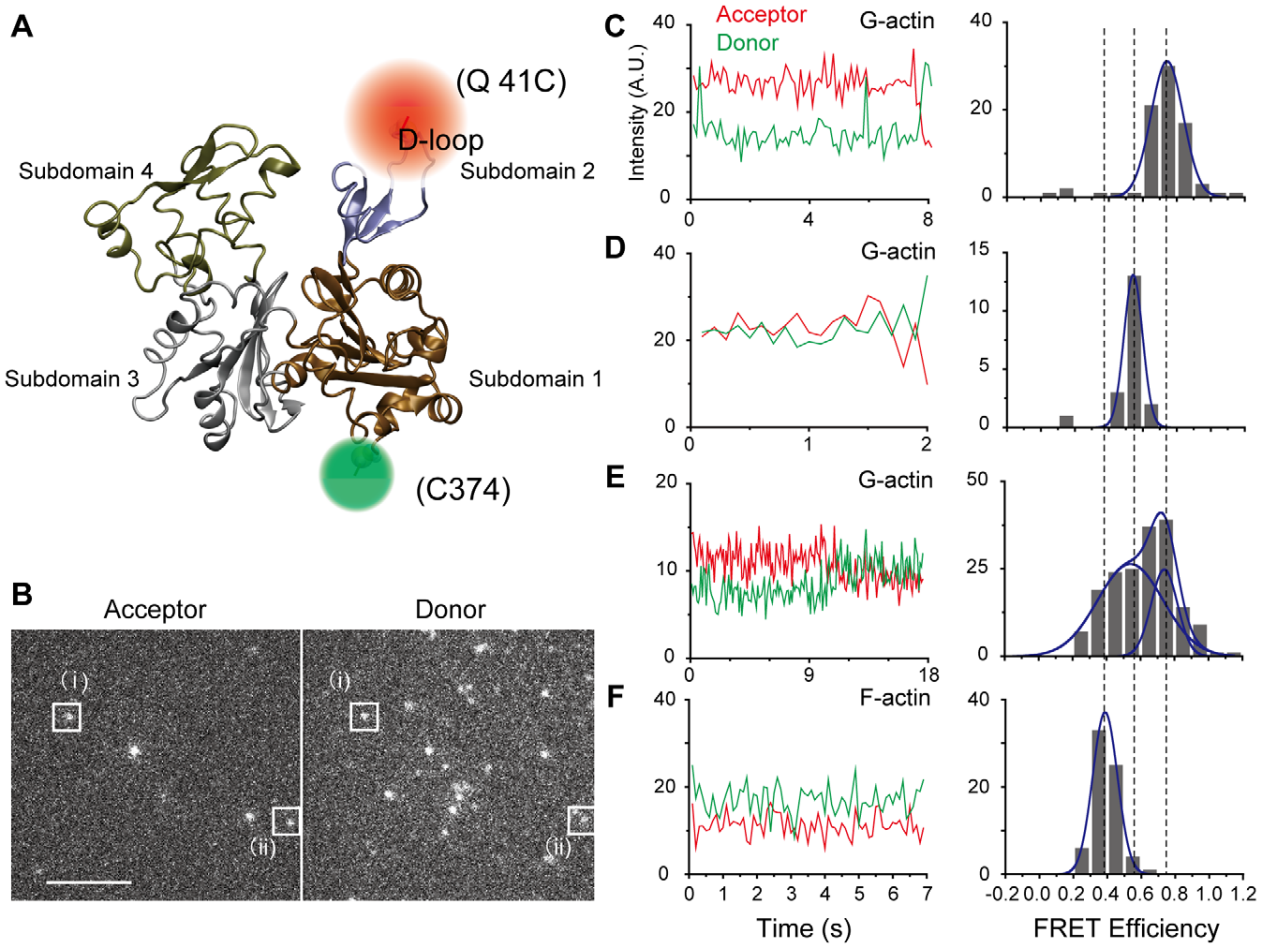
Individual actin has several structures. (A) G-actin has 4 subdomains. Residues 41 (D-loop region in subdomain 2) and 374 (C-terminal region in subdomain 1) were labeled with Alexa dyes of different wavelengths. (B) Fluorescent images of the acceptor (left images) and donor (right images) captured simultaneously by an electron multiplying charged coupled device camera. (i) and (ii) indicate the donors and acceptors for different individual actin molecules, respectively. The corresponding Förster resonance energy transfer (FRET) efficiencies of (i) and (ii) are shown in (C) and (D), respectively. The scale bar is 5 µm. (C–E) Time series of the acceptor and donor intensities from different single G-actin molecules and the corresponding FRET efficiency histograms. Two states can be seen. (F) Time series of the acceptor and donor intensities from a single F-actin molecule and the corresponding FRET efficiency histogram. A state distinct from that observed in G-actin can be seen.

We labeled the actin mutant Q41C at residues 41 and 374, which are located in subdomains 2 and 1, with Alexa 555(invitrogen) (donor) and Alexa 647(invitrogen) (acceptor) for FRET measurements [19] (Fig. 1A). These residues were chosen because their intermolecular cross-linking in adjacent protomers have been shown to affect both myosin motility [20] and the distance between them, which is sensitive to the binding of an ABP [21]. Fig. 1B shows fluorescent images of the acceptors (left panel) and donors (right panel) attached to individual G-actin and captured simultaneously by an electron multiplying charged coupled device (EMCCD) camera (Andor) with a dual-view apparatus (Hamamatsu Photonics) at 10 frames per second (fps) (see Materials and Methods and Figure S1). Figs. 1C and D show fluorescence intensities of the donors and acceptors attached to the single G-actins marked (i) and (ii) in Fig. 1B, respectively, and the corresponding histograms of their FRET efficiencies as calculated from a time series of the donor and acceptor intensities (Materials and Methods). The 2 molecules had different FRET efficiencies (∼0.8 and ∼0.5), indicating different conformational states. A third G-actin molecule showed a transition between these two states (Fig. 1E). The frequency of observing such transitions was low, however (11 of 312 observed molecules, or 3%), possibly because of the short observation time (average, ∼5 s) caused by photobleaching. Our calculations suggest a transition rate of 1/150 s^−1^ (5 s/150 s ∼0.03), which implies a long lifetime for the conformations (∼150 s).

**Figure 2:**
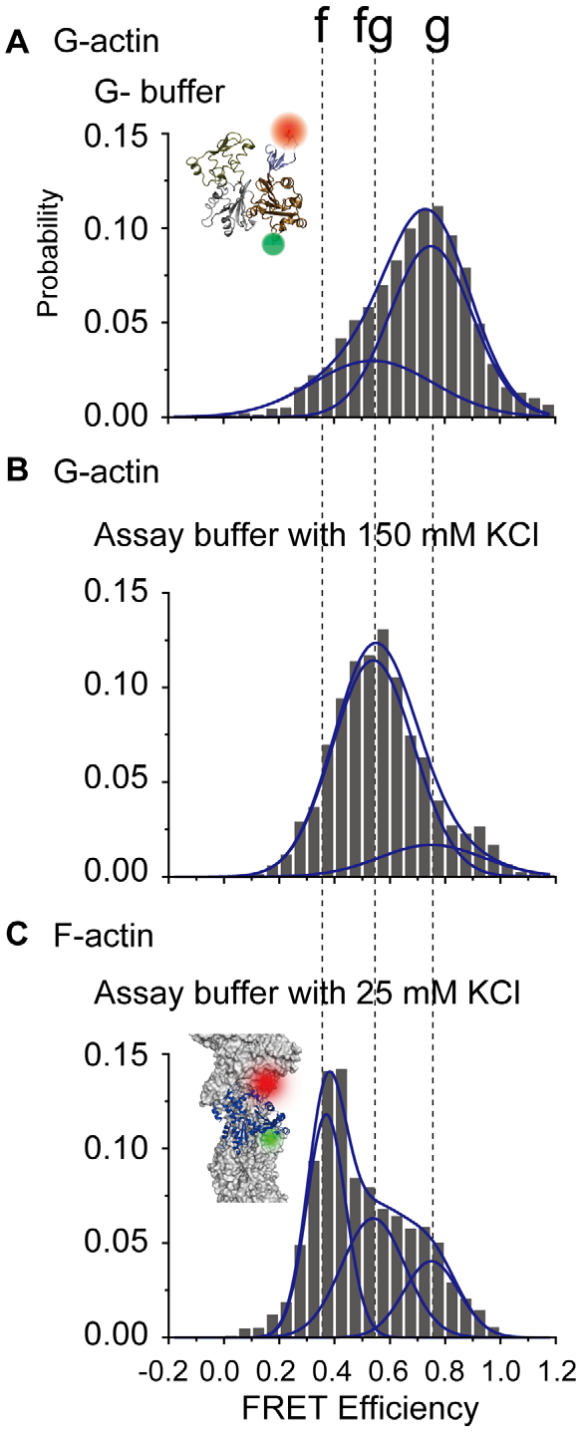
Distribution of G-and F-actin Förster resonance energy transfer (FRET) efficiencies for all observed molecules. (A,B) In the case of G-actin, samples were fixed onto a glass surface via an anti-myc antibody. Two states can be seen (g and fg) (cf. Fig. 1C–1E). Increasing the ionic strength increased the fg state. (C) In the case of F-actin, another state (f state) appeared. Peak positions were determined by fitting FRET distributions for all observed forms of actin to a sum of 2 or 3 Gaussian distributions (Figure S1). Peak positions for the f, fg, and g states are 0.37, 0.54, and 0.75, respectively. The numbers of molecules represented in the histograms are 312, 109, and 374 in (a), (b), and (c), respectively.

Histograms for FRET efficiencies summed over all samples are shown in Fig. 2. G-actin at low ionic strength is shown in Fig. 2A. The broad distribution could be roughly fit to 2 Gaussians with peaks at 0.75 and 0.54, which we define as the g and fg states, respectively. These multiple G-actin states are unlikely the result of actin polymerization because actin was fixed to the glass surface before the experiment. The peak values are consistent with the histograms shown in Figs. 1C–E. When calculating the average over both states, the average distance between the labeled residues was 4.4 nm (average FRET efficiency = 0.7, Materials and Methods), which is close to the value of the G-actin crystal structure (PDB ID: 1J6Z) [4]. When we applied 150 mM KCl solution (high ionic strength), creating a condition that promotes polymerization of G-actin if not affixed to the glass surface, the proportion of the fg state increased (Fig. 2B). This caused a change in the distance between the labeled residues (∼0.5 nm) (Materials and Methods), which was quite significant considering that the diameter of an actin monomer is only ∼5.4 nm. While it has been reported that the structure of the D-loop in subdomain 2 depends on nucleotide binding [22], [23], [24], this was not of concern here because we always used saturating ATP concentrations.

### Conformational states of F-actin

For F-actin, we mixed and copolymerized double-labeled actin, biotinylated actin, and non-labeled actin (rabbit skeletal muscle actin) at a ratio of 1∶10∶1000 in KCl (100 mM KCl, 5 mM MgCl_2_, and 2 mM EGTA) with phalloidin. F-actin was fixed onto a glass surface via streptavidin in assay buffer (20 mM Hepes-KOH [pH 7.8], 5 mM MgCl_2_, 1 mM EGTA) with 25 mM KCl. We checked the myosin motility of wild type F-actin and ∼50% double-labeled F-actin (Material and Methods) and concluded that the labeling had no significant effect on our experimental condition when the ratio of the double-labeled actin in F-actin protomer was low (∼0.1%). The FRET efficiencies of a single F-actin molecule showed a state (the f state) distinct from the fg and g states seen in G-actin (Fig. 1F). When FRET efficiencies were summed over all observed F-actin molecules, 3 distinct states emerged (Fig. 2C): the f state (0.37), the fg state (0.54), and the g state (0.75). The FRET distribution of F-actin was different from that of G-actin in the same buffer condition (Figure S2). The average distance between the labeled residues in F-actin was ∼5.1 nm (average FRET efficiency = 0.51), which was longer than that for G-actin. This may be due to differences in the D-loop conformation in subdomain 2, which is known to be more extended in F-actin than in G-actin [9]. Furthermore, similar to G-actin, increasing the ionic strength enhanced the fg state for F-actin (Figure S2).

## Discussion

**Figure 3:**
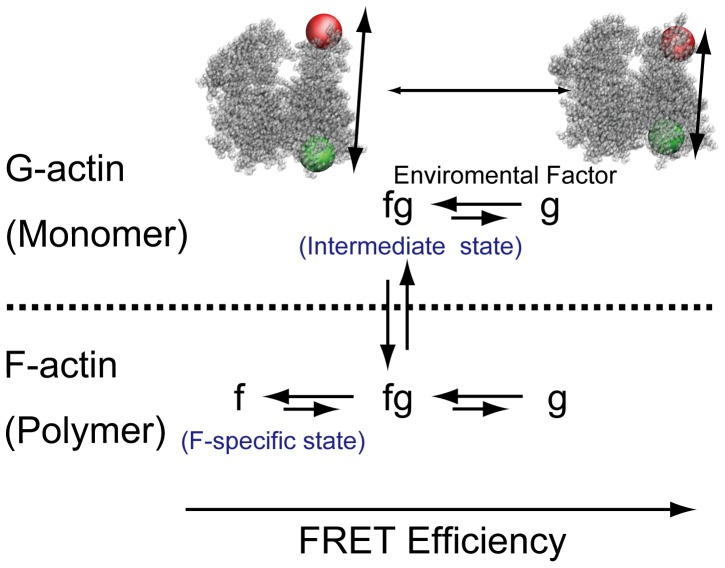
Model of actin structures based on the Förster resonance energy transfer (FRET) states. Using the FRET efficiencies, we surmise that the g and fg states in G-actin and F-actin are analogous. The f state appears only after polymerization and is, therefore, a specific state of F-actin.

On the basis of the FRET efficiencies, we surmised that the g and fg states in G-actin are analogous to the g and fg states in F-actin, while the f state is unique to F-actin (Fig. 3). Furthermore, at a high salt concentration, the fg state was increased. Considering the critical concentration for actin polymerization was reduced with increasing ionic strength, the results suggest that the critical concentration of actin polymerization varies with the ratio of the two G-actin states. Since the f state only exists in F-actin and high ionic strength enhances the fg state for both G- and F-actin (Figure S1), we considered that the G-actin in the fg state acts as an intermediary between G- and F-actin such that a protomer in this state likely attaches to the end of an actin filament and polymerizes. The existence of an intermediate state in G-actin is consistent with several reports that have implied the existence of putative protomer conformations just before polymerization [14], [15], [16]. Although flattening of the actin protomers (i.e., rotation of subdomains 1 and 2 relative to subdomains 3 and 4) regulates the transformation to F-actin [13], we have focused on a different part within subdomains 1 and 2 and observed distance changes there, which imply that another kind of conformational change may contribute to G-F transformation.

G-actin at a high ionic condition (physiological condition) constantly polymerizes and depolymerizes. G-actin monomers, which were non-specifically adhered to a glass surface, had a different FRET distribution than those that specifically adhered (Figure S3). G-actin attachment to the glass surface via an antibody allowed us to observe the structural states and to directly demonstrate the existence of intermediate actin protomers, which has been previously indicated [14], [15], [16], while avoiding polymerization and non-specific absorption (Figure S3). It has been reported that structural changes in the D-loop depend on the presence of cations [25], [26] and nucleotides [22], [23], [24]. In our experiments (Assay buffer with 25 mM KCl and 150 mM KCl), the distribution of the structural states depended on the KCl concentrations. Our results suggest that the residence time at a state are hundreds of seconds, whereas molecular dynamics simulations showed that structural changes in the D-loop occur on a scale of nanoseconds (Figure S5). Such fast structural changes cannot account for long intervals of transitions between multiple states (Text S1 and [24]). Since the ATP concentration in our experiments was maintained by using an ATP regeneration system, our observation is not the consequence of changes in the concentrations of ATP and ADP.

It was proposed that phalloidin stabilizes actin filaments, but does not directly attach to the position of the protomer to which we attached the dyes [27]. In the present experiments, it was crucial to avoid detection of FRET signals from G-actin monomers that were the result of depolymerization and non-specific adhesion to the glass surface (Figure S3). Therefore, we included phalloidin into the observation cell when observing F-actin. While there is the possibility that phalloidin changes the F-actin structures, we found that the ionic strength enhanced the fg state for F-actin independent of phalloidin (see Figure S4).

Our results showed multiple actin structures in the filaments. Recent cryo-EM studies have suggested structural models for F-actin; one of them showed a single well-defined structure [9], [10], and another suggested structural polymorphisms [11]. In cryo-EM experiments on F-actin, the sample filaments must be embedded on ice, which may bias and alter the distribution of the conformational states from those in solutions at room temperature. Hence, it is reasonable that the distribution of the states observed in our FRET experiments are different from those observed in cryo-EM conditions.

Overall, the distribution of multiple actin states depended on the ionic concentration and degree of polymerization. This finding is consistent with the notion that a high ionic concentration increases the intermediate state of G-actin likely leading to polymerization [14], [15], [16]. Our results suggest that actin undergoes spontaneous structural changes to enable G-F transformation, even without regulation by ABP. That is, polymerization of actin is modulated by the transition between multiple states, which are likely sensitive to the subcellular environment. We conjecture that the existence of multiple states enhances the flexibility of filaments and specific ABP binding, which may induce severing and branching [28]. Such transformations could be regulated by a mechanical distortion [29], [30] or by an intracellular crowding effect [31]. Actin may sense the local environment and change its state, which may alter the dynamics of the actin network in the cell and thus, contributes to the multifaceted role of actin in a number of cellular functions. Further understanding of these dynamical transitions should therefore provide new insight on cellular functions that depend on cytoskeletal dynamics.

## Materials and Methods

### Protein preparation

For G-actin experiments, rat mutated actin (Q41C) was recombined with myc-tag at the N-terminal position. Purification of mutated actin was done similarly to a previously described method [19]. Q41C actin (0.2 mg/ml) was mixed with Alexa 555 and 647 maleimide (Invitrogen), which can efficiently react with the S-H residues in cysteine, at a 1∶10 ratio and incubated for 8 h on ice for labeling. Unreacted dyes were removed by using AutoSeq G-50 columns (GE Healthcare). The labeling rate was ∼50%. By using this method, our sample contained 4 types of molecules (41-Alexa 555 and 374-Alexa 647, 41-Alexa 647 and 374-Alexa 555, 41-Alexa 555 and 374-Alexa 555, and 41-Alexa 647 and 374-Alexa 647). Intermolecular FRET is negligible because G-actin molecules were fixed onto the glass surface at a low density and the ratio of labeled actin protomers was low (∼0.1%) in the F-actin experiments, hence only the molecules with an Alexa 555 and an Alexa 647 were counted in our FRET measurements. There were still 2 types of molecules (41-Alexa 555 and 374-Alexa 647, 41-Alexa 647 and 374-Alexa 555). However, since the same samples were used for all conditions, changes in the FRET distributions cannot be accounted for by the existence of these 2 types of molecules in the sample.

For F-actin experiments, Q41C actin samples (0.2 mg/ml) were first labeled with Alexa 555 and Alexa 647 using the same method as for G-actin and copolymerized with biotinylated actin (Cytoskeleton) and non-labeled actin (rabbit skeletal muscle actin) at a 1∶10∶1000 ratio (total concentration was 1.0 mg/ml). Samples were incubated in a solution of high ionic strength (100 mM KCl, 5 mM MgCl_2_, and 2 mM EGTA) to accelerate actin polymerization within 30 min. We added phalloidin at a ratio of actin protomer∶phalloidin = 1∶1. Wild-type skeletal muscle actin and skeletal muscle myosin Subfragment 1 were purified from rabbit [32], [33]. We compared the motility of wild type skeletal actin (3.8±1.3 µm/s; [mean ± SD]) and ∼50% double-labeled F-actin (2.4±0.9 µm/s) and concluded that the labeling had no significant effect on the interaction with myosin (i.e., the labeled protomer is functional). In addition, since the ratio of the double-labeled actin in F-actin was low (∼0.1%), we concluded that the labeling induced no significant distortion of the filament in the experiments.

### Measurement system

A schematic diagram of the measurement system is shown in Figure S1. Single fluorescent molecule measurements [34] were performed with objective lens type TIRF microscopy on an inverted microscope (IX71; Olympus). Fluorescent dyes were excited with a 532-nm laser, which was rotated at 30 Hz using a piezo mirror in order to keep the polarization parallel with the circular trajectory and to rotate the direction of the evanescent wave polarization within the image plane [35], [36]. Wavelengths were separated by using several dichroic mirrors and emission filters in a dual view system (Hamamatsu Photonics). Data were acquired at 10 fps and analyzed. Photobleaching of the fluorescence spots confirmed that FRET occurred within single actin molecules. Only these spots were used for data analysis.

### Sample preparation

To fix the sample, a biotin–avidin system was used. Streptavidin were formed on a supported lipid bilayer constituted by lipid 1,2-dioleoyl-*sn*-glycero-3-phosphoethanolamine (DOPC, Avanti) and 1,2-dioleoyl-*sn*-glycero-3-phosphoethanolamine-*N*-(cap-biotinyl) (biotin-cap-DOPE, Avanti) (9∶1, weight ratio) [37]. Dried lipid films were obtained by mixing appropriate amounts of the lipids dissolved in chloroform followed by evaporating the solvent for 6 h. To obtain multilamellar vesicles, the dried lipid films were resuspended by vortexing in 20 mM Tris-HCl (pH 7.8). Small unilamellar vesicles were produced by sonication with a tip-sonicator and separated from the multilamellar vesicles by ultracentrifugation. Small unilamellar vesicles were coated on the cover glass surface with 2 mM CaCl_2_.

G-actin samples (1 nM) were fixed onto a glass surface using an anti-biotinylated myc-tag antibody (Clontech and Dojindo biotinylation kit) via biotin-lipid bilayer and streptavidin (2.0 mg/ml). Non-adsorbed actin was removed by washing with G-buffer (2 mM Hepes-KOH [pH 7.8], 0.2 mM CaCl_2_, and 0.2 mM ATP). After washing, the buffer was substituted with G-buffer or assay-buffer (20 mM Hepes-KOH [pH 7.8], 5 mM MgCl_2_, 1 mM EGTA) with 25 mM or 150 mM KCl, respectively.

F-actin samples were adsorbed onto the glass surface via biotin-lipid bilayer and streptavidin and measured with 10 µM phalloidin. All experiments were performed with an oxygen scavenger system [38] and 100 mM dithiothreitol (DTT) at 26°C. In those experiments that included ATP, 1 mM ATP and an ATP regeneration system (2 U/ml creatine kinase and 2 mM creatine phosphate) were added to the system.

### Analysis of FRET data

Single molecule FRET was used to reveal dynamic structural changes in actin. FRET efficiency, *E*, was measured as 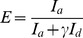, where *I_a_* and *I_d_* are the acceptor and donor fluorescent intensities, respectively, and 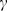 is the correction factor of the fluorescence and obtained as described below. The donor signal recovery after acceptor photobleaching is related to the quantum yields of the fluorescent molecules and the overall detection efficiency. Fluorescence intensity data were acquired through a moving average window of over 1 s and analyzed by using LabVIEW. The 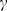 value is equivalent to |*ΔI_a_/ΔI_d_*|, where Δ*I_a_* and Δ*I_d_* are the changes of the acceptor and donor intensities upon acceptor photobleaching, respectively, and calculated from the average intensity of the donor and acceptor before and after photobleaching in each experiment. The median of 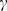 in each condition (shown in Table S1) was used for the analysis. The distance between the 2 dyes was determined by 
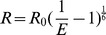
 (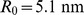) when neglecting the size and dipole direction of the dyes. More detailed quantitative analysis is shown in SI.

## Supporting Information

Figure S1 **Schematic diagram of the experimental setup.**
(TIF)

Figure S2 **Distribution of G and F-actin Förster resonance energy transfer (FRET) efficiencies.** Additional data (not shown in Fig. 2) is shown. Color codes correspond to the classification in the statistical analysis shown in Text S1 and Table S1.
(TIF)

Figure S3 **Comparisons of non-specific adhesion of G-actin to the glass surface via an anti-myc-antibody in assay buffer with 150 mM KCl.** Distribution of the FRET efficiencies in non-specifically adhered G-actin is shown. (cf. Fig. 2B). Non-specific adhesion increased the probability of the g state, as compared to the specifically binding case using antibody.
(TIF)

Figure S4 **The effects of phalloidin on F-actin.** Distribution of the FRET efficiencies in F-actin without phalloidin is shown (cf. Fig. 2C). High ionic strength increased the fg state of F-actin. In the absence of phalloidin, G-actin depolymerized from the filament and adhered to the glass surface non-specifically, affecting the distribution of its states.
(TIF)

Figure S5 **Time series of the corresponding distance observed in molecular dynamics simulations.** The distance between the α-carbon atoms in residues 41 and 374 is shown. (G) an isolated G-actin monomer and (F) an F-actin model (pentamer).
(TIF)

Table S1 **Distribution of Förster resonance energy transfer (FRET) states in each condition.**
(DOC)

Text S1 **Details of statistical analysis of the Förster resonance energy transfer (FRET) states and molecular dynamics simulations.**
(DOCX)
