# Supplementary figures and images for: Spontaneous Structural Changes in Actin Regulate G-F Transformation
Source: PLoS One. 2012 Nov 5;7(11):e45864. doi: 10.1371/journal.pone.0045864 (PMC3489878; doi:10.1371/journal.pone.0045864)

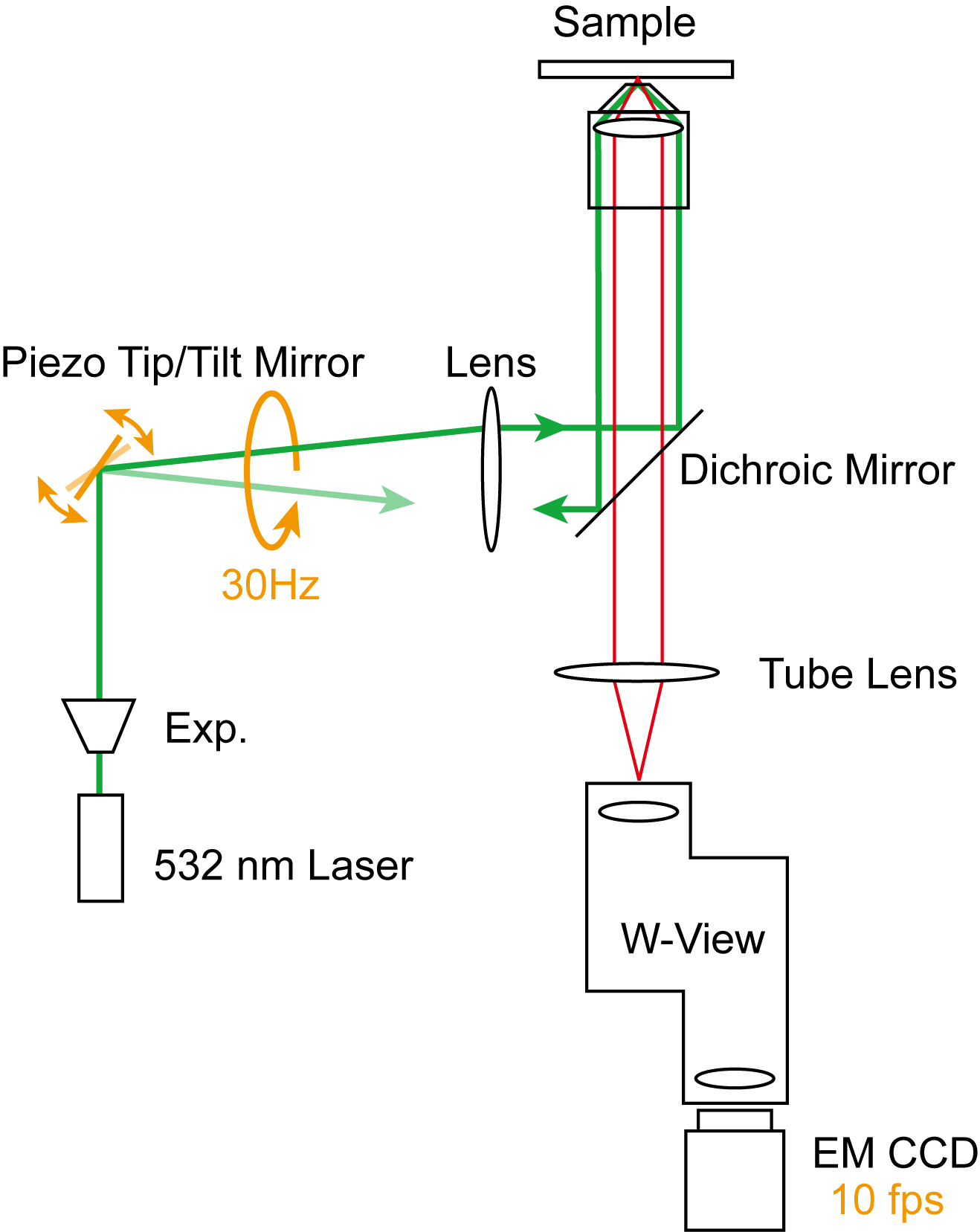

Supplement: Figure S1 — Schematic diagram of the experimental setup. (TIF) [file pone.0045864.s001.tif]

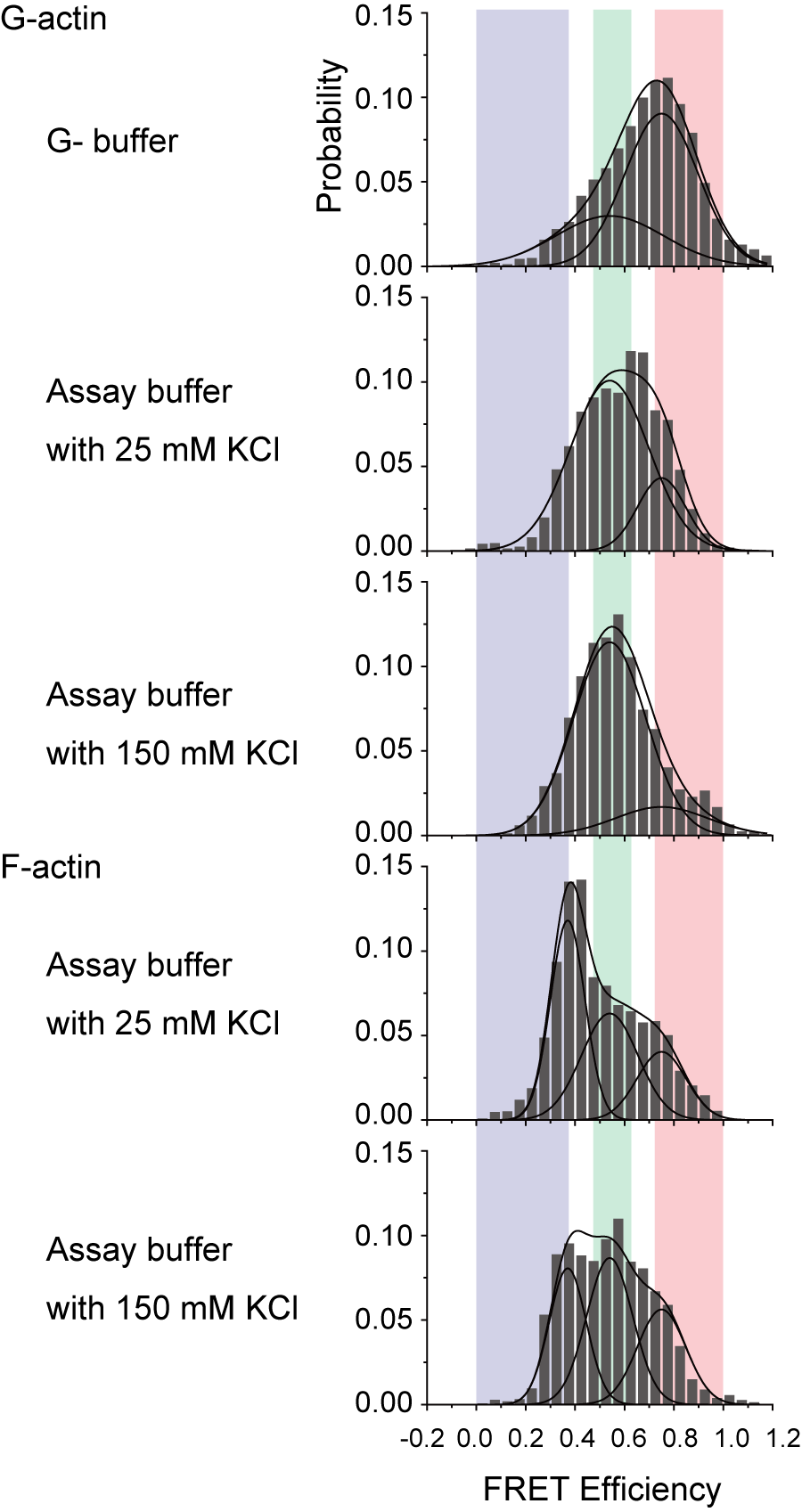

Supplement: Figure S2 — Distribution of G and F-actin Förster resonance energy transfer (FRET) efficiencies. Additional data (not shown in Fig. 2) is shown. Color codes correspond to the classification in the statistical analysis shown in Text S1 and Table S1. (TIF) [file pone.0045864.s002.tif]

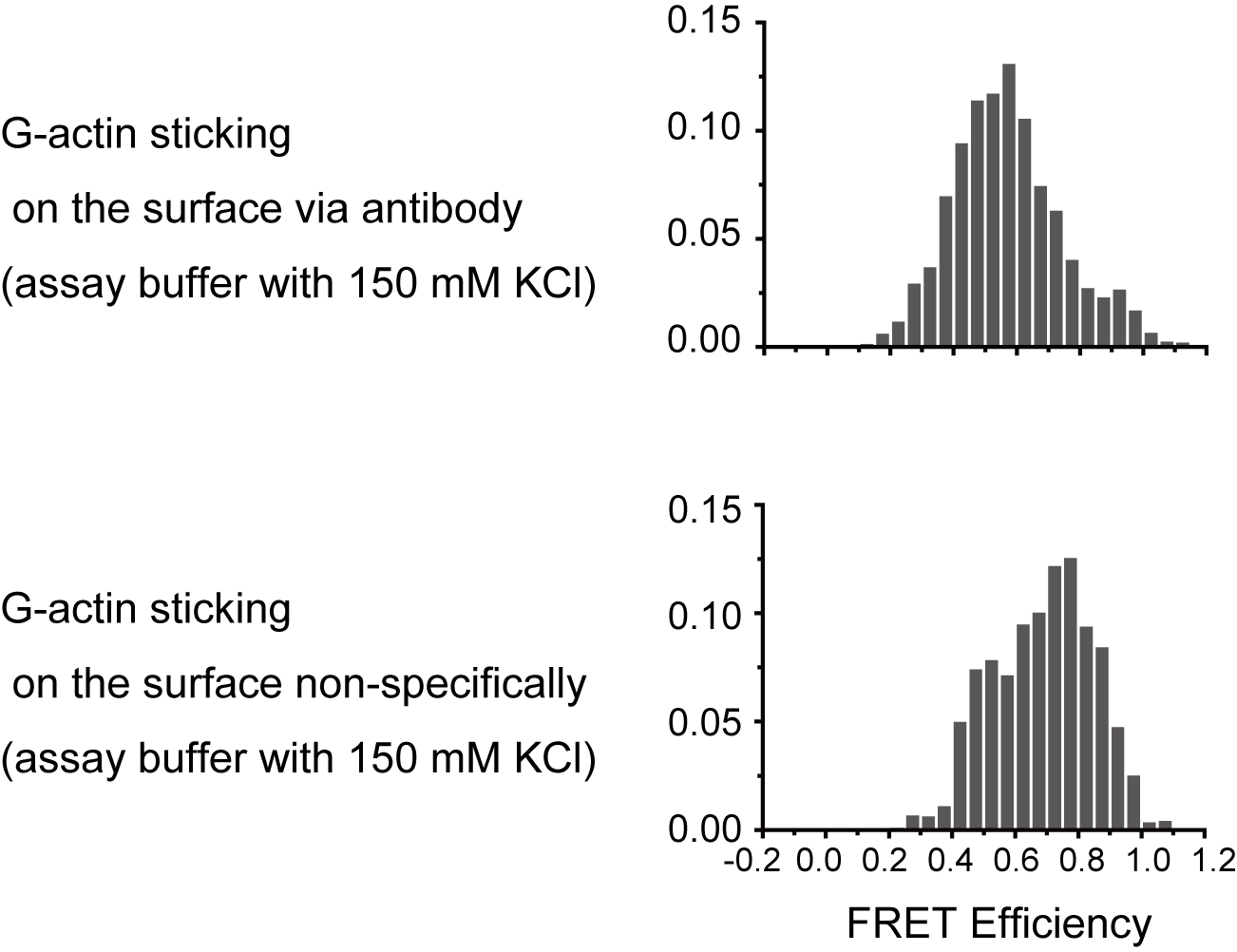

Supplement: Figure S3 — Comparisons of non-specific adhesion of G-actin to the glass surface via an anti-myc-antibody in assay buffer with 150 mM KCl. Distribution of the FRET efficiencies in non-specifically adhered G-actin is shown. (cf. Fig. 2B). Non-specific adhesion increased the probability of the g state, as compared to the specifically binding case using antibody. (TIF) [file pone.0045864.s003.tif]

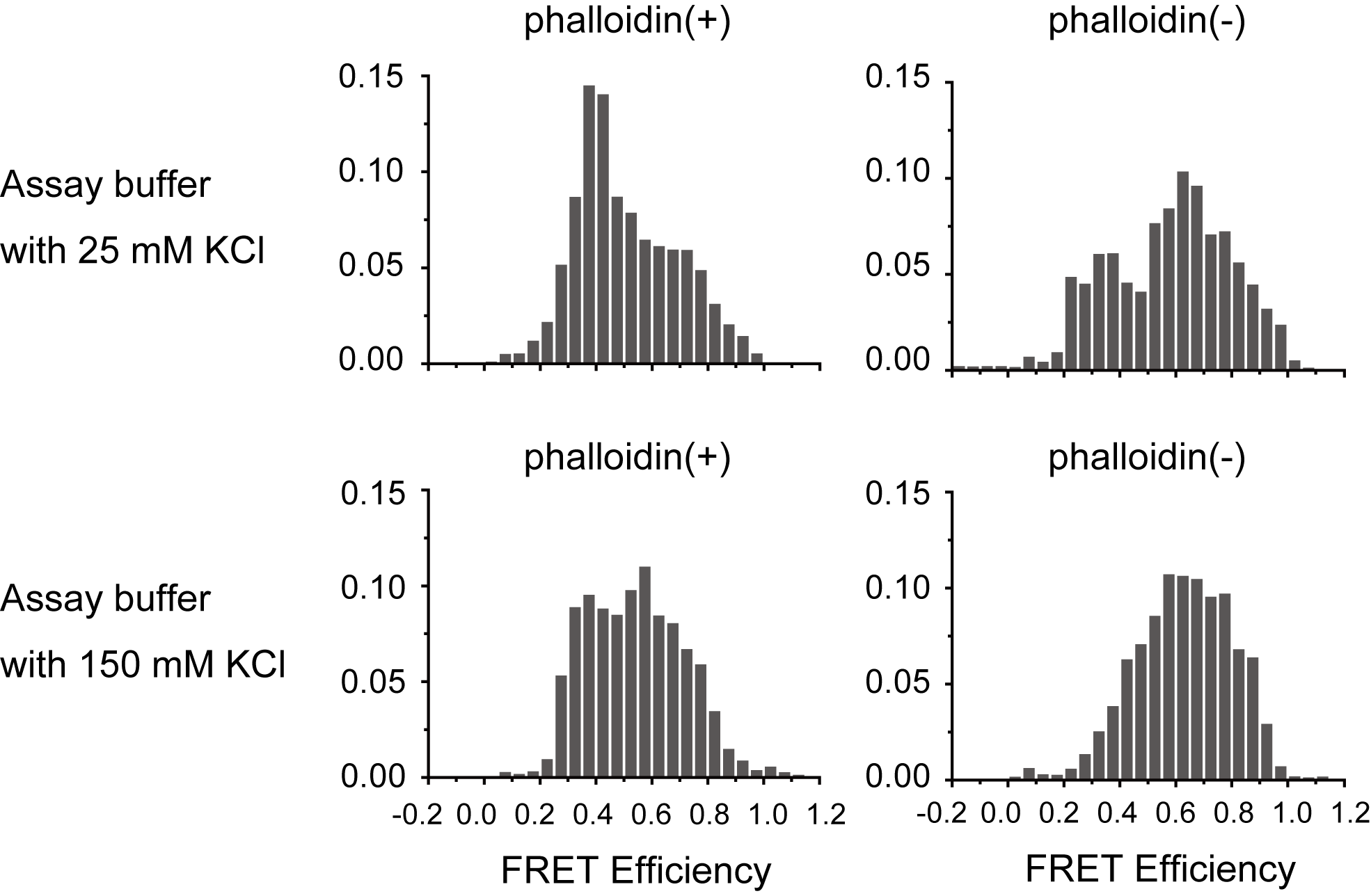

Supplement: Figure S4 — The effects of phalloidin on F-actin. Distribution of the FRET efficiencies in F-actin without phalloidin is shown (cf. Fig. 2C). High ionic strength increased the fg state of F-actin. In the absence of phalloidin, G-actin depolymerized from the filament and adhered to the glass surface non-specifically, affecting the distribution of its states. (TIF) [file pone.0045864.s004.tif]

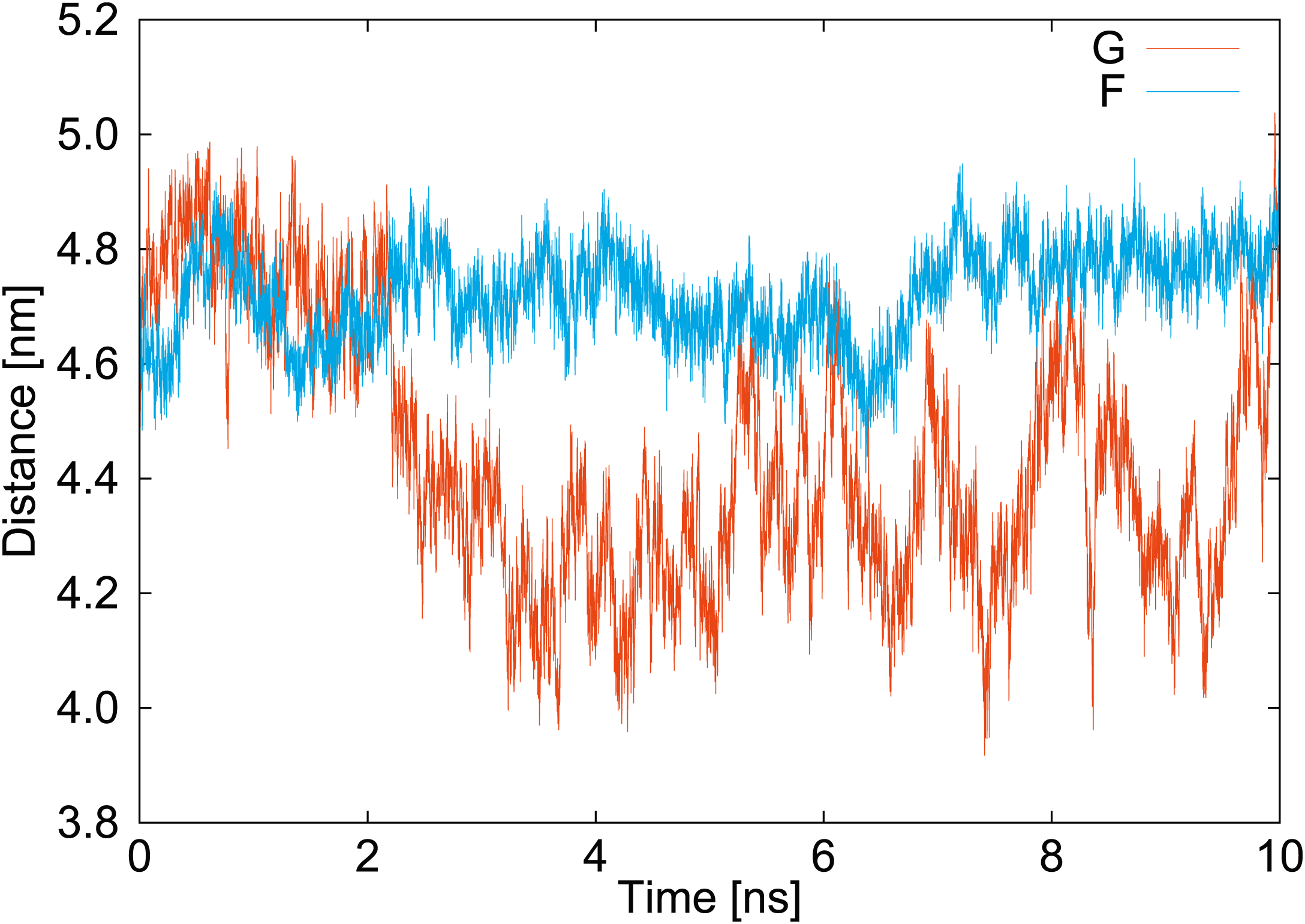

Supplement: Figure S5 — Time series of the corresponding distance observed in molecular dynamics simulations. The distance between the α-carbon atoms in residues 41 and 374 is shown. (G) an isolated G-actin monomer and (F) an F-actin model (pentamer). (TIF) [file pone.0045864.s005.tif]
