# Supplementary material for: Spontaneous Structural Changes in Actin Regulate G-F Transformation
Source: PLoS One. 2012 Nov 5;7(11):e45864. doi: 10.1371/journal.pone.0045864 (PMC3489878; doi:10.1371/journal.pone.0045864)
Supplement: Table S1 — Distribution of Förster resonance energy transfer (FRET) states in each condition. (DOC) [file pone.0045864.s006.doc]

Table S1: Distribution of Förster resonance energy transfer (FRET) states in each condition

(a) Measurement conditions, numbers of samples, and $\gamma$ used in the analysis.

| Condition | Actin | Solution | Number of molecules | $\gamma$ (median) | Number of data points | |
| --- | --- | --- | --- | --- | --- | --- |
|  |  |  |  |  | $0<E\leq1$ | All valid |
| GG | G-actin | G-buffer | 312 | 1.3 | 14965 | 15963 |
| G25 |  | 25 mM KCl | 135 | 1.1 | 4730 | 4782 |
| G150 |  | 150 mM KCl | 109 | 1.1 | 4398 | 4450 |
| F25 | F-actin | 25 mM KCl | 374 | 0.87 | 10274 | 10280 |
| F150 |  | 150 mM KCl | 337 | 1.0 | 14892 | 15074 |

(b) Statistical test for low, medium and high FRET populations.

|  | $N_{i}$ | $E\leq0.375$ proportion | $P'$-values against | | | |
| --- | --- | --- | --- | --- | --- | --- |
|  |  |  | G25 | G150 | F25 | F150 |
| GG | 312 | 0.06816 | 0.042 | 0.096 |  |  |
| G25 | 135 | 0.11987 |  | >0.5 | 0.0087 |  |
| G150 | 109 | 0.11210 |  |  |  | 0.018 |
| F25 | 374 | 0.21608 |  |  | | >0.5 |
| F150 | 337 | 0.20615 |  |  | | |

|  | $N_{i}$ | $0.475<E\leq0.625$ proportion | $P'$-values against | | | |
| --- | --- | --- | --- | --- | --- | --- |
|  |  |  | G25 | G150 | F25 | F150 |
| GG | 312 | 0.20708 | 0.023 | 0.00027 |  |  |
| G25 | 135 | 0.29598 |  | 0.14 | 0.071 |  |
| G150 | 109 | 0.37199 |  |  |  | 0.13 |
| F25 | 374 | 0.22698 |  |  | | 0.011 |
| F150 | 337 | 0.30271 |  |  | | |

|  | $N_{i}$ | $0.725<E$ proportion | $P'$-values against | | | |
| --- | --- | --- | --- | --- | --- | --- |
|  |  |  | G25 | G150 | F25 | F150 |
| GG | 312 | 0.43856 | 9.0×10^-7^ | 1.6×10^-7^ |  |  |
| G25 | 135 | 0.20465 |  | 0.36 | 0.18 |  |
| G150 | 109 | 0.16735 |  |  |  | >0.5 |
| F25 | 374 | 0.16099 |  |  | | >0.5 |
| F150 | 337 | 0.16190 |  |  | | |

(Blue: 1%, Green: 5% significance)
