# Supplementary material for: Spontaneous Structural Changes in Actin Regulate G-F Transformation
Source: PLoS One. 2012 Nov 5;7(11):e45864. doi: 10.1371/journal.pone.0045864 (PMC3489878; doi:10.1371/journal.pone.0045864)
Supplement: Text S1 — Details of statistical analysis of the Förster resonance energy transfer (FRET) states and molecular dynamics simulations. (DOCX) [file pone.0045864.s007.docx]

**Text S1**

**Proportion of Conformational States: Statistical Analysis**

In Figs. 2 and 3, we assumed three conformational states (f, fg, and g), and estimated the proportion of these states by fitting the FRET distribution by the sum of three corresponding Gaussian distributions using the least-squares method. The estimation is based on the three-state model, and may contain some fitting errors. To be more precise, here we introduce a stricter measure for the changes of the FRET-state distribution, which does not depend on any assumptions about the number and FRET-distribution of states, and determine the lower bound of the proportion of affected protomers. In the analysis below, we used only data points with FRET efficiency$0<E\leq1$, to eliminate erroneous data.

First, we set the threshold value $E_{0}$ for FRET efficiency. Each data point with FRET efficiency $E$ was classified as either $E\leq E_{0}$ or $E>E_{0}$. The proportion of $E\leq E_{0}$ points was denoted by $q$ below. Suppose that $q=q_{1}$ in one condition and $q=q_{2}$ in another, and that $q_{1}<q_{2}$. In a large ensemble, the FRET efficiency of at least $(q_{2}-q_{1})$ of total samples must be altered, in which case all the changes are unidirectional across the threshold$E_{0}$, to attain the difference in the proportion. Thus, $\Delta q=(q_{2}-q_{1})$ represents the lower limit of the proportion of molecules affected by a change in conditions. It should be noted that $E_{0}$ is arbitrary and thus we can set $E_{0}$ to maximize $\Delta q$.

Of course, since the samples are finite, we may argue that $\Delta q$ is overestimated due to fluctuations. To refute this, we estimated the lower limit of $\Delta q$ with a certain confidence level as follows. Suppose that we observe $q=q_{1}$ and $q_{2}$ from $N_{1}$ and $N_{2}$ independent samples in each condition ($q_{1}<q_{2}$), and that the true $q$ values are $\tilde{q}_{1}$ and $\tilde{q}_{2}$, respectively. $N_{i}q_{i}\text{ (}i=1,\text{ }2\text{)}$, i.e. the number of observed $E\leq E_{0}$ samples, obeys the binomial distribution $B(N_{i},\tilde{q}_{i})$. Assuming that $N_{i}\tilde{q}_{i}$ is sufficiently large, $B(N_{i},\tilde{q}_{i})$ is approximated by a Gaussian distribution with the same mean and variance as the binomial such that,
.
Then, the probability of observing $q\leq q_{1}$ in condition 1 and $q\geq q_{2}$ in condition 2 is:
,
which represents the level of significance ($P=1/4$ if ; to be fair, we use $P'=4P$ instead of $P$ below). $\Delta q_{\min(\alpha)}$, the lower bound of $\Delta q$ at the confidence level $\alpha$, is the maximum value of that satisfies . We scanned $\Delta\tilde{q}$ and $\tilde{q}_{1}$ numerically to obtain $\Delta q_{\min(\alpha)}$.

There may exist in our experiments correlations between data points in a time series from a single FRET pair. To eliminate any assumptions about the correlation time, we assumed the number of independent samples to be $N_{i}$, the number of molecules. Note that it is the worst case where all data points in a time series are perfectly correlated; actual $P'$ value may be much smaller, i.e. the change is statistically more significant, than calculated here. Also, the actual number of data points is much larger than $N_{i}$, hence the approximation of a binomial as Gaussian is even more appropriate than it is above.

For G-actin in G-buffer and assay buffer with 150 mM KCl, and $E{}_{0}=0.725$, $\alpha=0.95$, we obtained $\Delta q_{\min(0.95)}=0.21884$, where $N_{1}=312,\text{ }q_{1}=0.43856$ in the former and $N_{2}=109,\text{ }q_{2}=0.16735$ in the latter condition ($P'$ is maximum at ). Hence, in this case, at least 21.8% of the G-actin population were affected at 95% confidence level (cf. 27.1% if we simply calculate $q_{1}-q_{2}$).

Similarly, by setting $\Delta\tilde{q}=0$ and finding the maximum value of $P'$, we can test the null hypothesis: the true proportion $\tilde{q}$ is equal in the two conditions (). We applied this test to the observed proportion of $E\leq0.375$ (low FRET), $0.475<E\leq0.625$ (medium FRET) and $0.725<E$ (high FRET) samples (colored in Fig. 2). In Supplementary Table 1, we show the $P'$-values, which indicate significant effects by KCl both on G-actin (increase of low and medium FRET, and decrease of high FRET) and F-actin (increase of medium FRET).

**Simulations of F- and G- actin Molecular Dynamics**

The structure and initial atomic coordinates of actin were taken from one of the actin filament models in ref. [1] (Protein Data Bank ID code 3B5U). To simulate the dynamics of a single protomer in a filament (F-actin), we constructed a pentamer using 5 subsequent protomers (chains E to I) from the structural data as a minimal model, and observed the behavior of the protomer in the middle, i.e., chain G. For comparison, we also modeled an isolated monomer (G-actin) using the same structural data for chain G.

We used NAMD (version 2.7b1) [2] for our molecular dynamics simulations. The CHARMM22/CMAP all atom force field [2,3] was adopted with the TIP3P explicit water model and periodic boundary condition. Using VMD (version 1.8.6) [4], we supplemented hydrogen atoms, placed the model into a water box, and added K^+^ ions to cancel the negative charge of the actin molecules (13 ions per protomer). The dimensions of the box were ca. 12×12×21 nm^3^ (including 85926 water molecules) for the F-actin model and ca. 9×9×9 nm^3^ (including 20803 water molecules) for G-actin.

We applied the Particle Mesh Ewald method. The cutoff distance for the direct calculation was 12 Å. Van der Waals interactions were truncated at 12 Å with a switch function starting from 10 Å. We minimized the energy for 1000 steps and then integrated at the time step of 2 fs (4 fs for full electrostatics evaluation) with the SHAKE algorithm. The temperature and pressure were kept at 300 K and 1 atm by using Langevin thermostat (damping coefficient 5 ps^-1^) and Langevin piston Nose-Hoover method (period 100 fs, damping 50 fs), respectively.

Supporting Online Materials Figure 2 shows the first 10 ns of the distance between the α-carbon atoms in residues 41 and 374 to which fluorescent labels are attached for the FRET experiments. The distance shows large fluctuations in the isolated G-actin monomer, while such fluctuations are restricted by the interactions between adjacent protomers in the F-actin model. Snapshots of the conformations are shown in Supporting Online Materials Figure 3. The simulations show that ~1 nm changes of the difference, observed in the experiments, is plausible.

**Note: Simulations of G- and F-actin molecular dynamics**

Figure S5 shows time series of the distance between the α-carbon atoms in residues 41 and 374, to which fluorescent labels were attached for the FRET experiments, for the first 10 ns. The distance shows large fluctuations (~1 nm) in the isolated G-actin monomer, mainly due to high flexibility of the D-loop. Such fluctuations are restricted by the interactions between adjacent protomers in the F-actin model.

In our FRET experiments, the time resolution was much longer (~0.1 s) and the fluctuations at nano- to microsecond scales were averaged out. Nevertheless, we have observed large differences (also ~1 nm) in the distances between the f and g states. Conformational biases on the D-loop cannot fully account for the transitions between these states, especially in F-actin. We conjecture that the transitions involve conformational changes in stiffer parts inside the molecule, which may account for the long intervals between the transitions.

**Acknowledgments**

NAMD and VMD were developed by the Theoretical and Computational Biophysics Group in the Beckman Institute for Advanced Science and Technology at the University of Illinois at Urbana-Champaign [2,5].

[1] Y. Cong, M. Topf, A. Sali, P. Matsudaira, M. Dougherty, W. Chiu, and M. F. Schmid, Crystallographic conformers of actin in a biologically active bundle of filaments, J. Mol. Biol., 375, 331-336 (2007).

[2] J. C. Phillips, R. Braun, W. Wang, J. Gumbart, E. Tajkhorshid, E. Villa, C. Chipot, R. D. Skeel, L. Kale, and K. Schulten, Scalable molecular dynamics with NAMD, J. Comput. Chem., 26, 1781-1802 (2005).

[3] A. D. MacKerell, Jr., D. Bashford, M. Bellott, R. L. Dunbrack, Jr., J. D. Evanseck, et al., All-atom empirical potential for molecular modeling and dynamics studies of proteins, J. Phys. Chem. B, 102, 3586-3616 (1998).

[4] A. D. MacKerell, Jr., M. Feig, C. L. Brooks III, Extending the treatment of backbone energetics in protein force fields: limitations of gas-phase quantum mechanics in reproducing protein conformational distributions in molecular dynamics simulations, J. Comput. Chem., 25, 1400-1415 (2004).

[5] W. Humphrey, A. Dalke, and K. Schulten, VMD - Visual Molecular Dynamics, J. Molec. Graphics, 14, 33-38 (1996).
